# Volumetric MRI Markers and Predictors of Disease Activity in Early Multiple Sclerosis: A Longitudinal Cohort Study

**DOI:** 10.1371/journal.pone.0050101

**Published:** 2012-11-15

**Authors:** Tomas Kalincik, Manuela Vaneckova, Michaela Tyblova, Jan Krasensky, Zdenek Seidl, Eva Havrdova, Dana Horakova

**Affiliations:** 1 Department of Neurology and Center of Clinical Neuroscience, 1st Faculty of Medicine, Charles University in Prague and General University Hospital, Prague, Czech Republic; 2 Melbourne Brain Centre, Department of Medicine, University of Melbourne, Melbourne, Australia; 3 Department of Radiology, 1st Faculty of Medicine, Charles University in Prague and General University Hospital, Prague, Czech Republic; University of Maryland, College Park, United States of America

## Abstract

**Objectives:**

To compare clinical and MRI parameters between patients with clinically isolated syndrome and those converting to clinically definite multiple sclerosis within 2 years, to identify volumetric MRI predictors of this conversion and to assess effect of early relapses.

**Methods:**

The SET study comprised 220 patients with clinically isolated syndrome treated with interferon beta (mean age, 29 years; Expanded Disability Status Scale, 1.5). Three patients with missing data were excluded from the analysis. Physical disability, time to clinically definite multiple sclerosis and volumetric MRI data were recorded for 2 years.

**Results:**

Patients reaching clinically definite multiple sclerosis showed impaired recovery of neurological function, faster decrease in corpus callosum cross-sectional area, higher T2 lesion volume and more contrast-enhancing lesions. Six-month decrease in corpus callosum cross-sectional area (≥1%) and baseline T2 lesion volume (≥5 cm^3^) predicted clinically definite multiple sclerosis within 2 years (hazard ratios 2.5 and 1.8, respectively). Of 22 patients fulfilling both predictive criteria, 83% reached clinically definite multiple sclerosis (hazard ratio 6.5). More relapses were associated with poorer recovery of neurological function and accelerated brain atrophy.

**Conclusions:**

Neurological impairment is more permanent, brain atrophy is accelerated and focal inflammatory activity is greater in patients converting to clinically definite multiple sclerosis. Six-month corpus callosum atrophy and baseline T2 lesion volume jointly help predict individual risk of clinically definite multiple sclerosis. Early relapses contribute to permanent damage of the central nervous system.

## Introduction

Approximately one half of patients with clinically isolated syndrome (CIS) develop clinically definite multiple sclerosis (CDMS) within the initial 5 years of the first clinical relapse and this proportion grows to two thirds at 20 years.[1]–[4] It has been shown that disease modifying therapy has the potential to delay a second demyelinating event and thus decrease the CDMS conversion rate. [5] Therefore, early identification of patients at high risk of ongoing relapsing activity is crucial for timely treatment escalation.

Structural changes within the central nervous system (CNS) occur early in multiple sclerosis (MS) course. [6] Abnormal brain MRI at the time of CIS is associated with increased risk of further relapses, i.e. of CDMS conversion. [7], [8] Both localised inflammatory changes and the rate of brain atrophy can predict risk of ongoing relapsing activity. [2], [4]

Here we report outcomes of the 2-year analysis of longitudinal quantitative MRI data from the SET study (Study of Early Interferon β1-a Treatment in High Risk Subjects after CIS). The SET cohort comprises 220 patients with CIS confirmed by positive MRI and cerebrospinal fluid (CSF) findings. Our aims were (i) to compare clinical and MRI changes between patients with CIS and those converting to CDMS, (ii) to identify early MRI predictors of CDMS conversion and (iii) to evaluate the effect of relapses on clinical and MRI parameters.

## Patients and Methods

### Ethics Statement

This study was approved by the Medical Ethics Committees of the General University Hospital and 1^st^ Faculty of Medicine, Charles University in Prague, and by local medical ethics committees in all participating centres. Written informed consent was obtained from every participant upon enrolment. The study was conducted according to the principles expressed in the Declaration of Helsinki.

### Study Design and Population

The SET (Study of Early Interferon β1-a Treatment in High Risk Subjects after CIS; EudraCT identification number 2005-001281-13) is an investigator-initiated, multi-centre, prospective observational study investigating clinical and MRI predictors of response to interferon β-1a (Avonex 30 µg IM weekly; Biogen Idec, Weston, MA, USA) therapy in CIS. Subjects were recruited from 8 MS centres within the Czech Republic (General University Hospital, Praha; KZ Hospital, Teplice; University Hospitals in Brno, Plzen and Olomouc; St. Anne’s University Hospital, Brno; Motol University Hospital, Praha and Kralovske Vinohrady University Hospital, Praha) between years 2005 and 2009. The study database was locked in August 2011.

Study inclusion criteria were: 18–55 years of age, enrolment within 4 months of the first clinical demyelinating event, Expanded Disability Status Scale (EDSS) score ≤3.5, presence of ≥2 hyperintense T2 lesions prior to steroid treatment and ≥2 oligoclonal bands in the CSF.* Exclusion criteria were: second relapse before enrolment, any major disease or pregnancy. Of 220 patients recruited, longitudinal data from 217 participants were analysed, with data from 3 patients excluded for incompleteness. The female:male ratio was 145∶72, with a mean age 29±8 years and median EDSS (interquartile range) 1.5 (1.5, 2).

After the screening procedure (comprising clinical examination, diagnostic MRI and lumbar puncture), patients were treated with 3–5 g of IV methylprednisolone (Solu-Medrol; Pfizer, Praha, Czech Republic). Baseline MRI was performed at least 30 days later. Within 4 months of the initial episode, patients’ disability was scored using EDSS and Multiple Sclerosis Functional Composite (MSFC) and they commenced treatment with interferon β-1a (Avonex 30 µg IM weekly).

### Treatment

One hundred and seventy patients (78%) remained on the assigned treatment throughout the 2-year follow-up, whereas 28 switched to another interferon preparation [Rebif 22 µg (2) or 44 µg (26) SC 3/week; Merck Serono, Geneva, Switzerland], 5 participants switched to glatiramer acetate (Copaxone 20 mg SC daily; Teva Pharmaceuticals, Petach Tikva, Israel), 2 escalated to natalizumab (Tysabri 300 mg IV monthly; Biogen Idec) and 12 discontinued disease modifying treatment. In addition to the disease modifying treatment, 17 patients received azathioprine (Azaprine 50 mg PO 2/day; Teva Pharmaceuticals) 1 patient received mitoxantrone (Refador 10 mg; Teva Pharmaceuticals) with 1 g of methylprednisolone IV monthly over 3 months and 8 patients received oral prednisolone (Prednison 5–10 mg PO daily; Zentiva, Praha, Czech Republic) or methylprednisolone (Medrol 8 mg PO daily; Pfizer). These treatment changes were made in accordance with the SET study protocol, in patients showing insufficient treatment effect (i.e. 2 moderate relapses or 6-month sustained progression of 1 EDSS step during 12 months on treatment) or lack of tolerance (unacceptable flu-like symptoms despite symptomatic treatment or 3-fold increase in liver enzyme concentrations).

### Clinical Evaluation

After the assessment of baseline disability and treatment initiation, patients were followed up in 3-month intervals, with disability rated at 6, 12, 18 and 24 months. EDSS scores were evaluated by accredited raters at each of the participating centres. Components of MSFC (timed 25-foot walk, 9-hole peg test and paced auditory serial addition test) were administered and evaluated by trained research assistants at every centre.

### MRI Acquisition and Analysis

Brain MR images were obtained on a 1.5T scanner (Gyroscan, Philips Medical Systems, Best, The Netherlands) at months 0, 6, 12 and 24 as described elsewhere. [9] Axial brain images were acquired using fast fluid-attenuated inversion recovery (FLAIR; TE/TR/TI: 140/11000/2600 ms, matrix size 256×181, flip angle 90°, slice thickness 1.5/0 mm, field of view = 256 mm) and T1-weighted 3-dimensional fast field echo images (TE/TR: 5/25 ms, flip angle = 30°, matrix size 256×256, slice thickness = 1/0 mm, field of view = 256 mm). Post-contrast T1-weighted images (TE/TR = 11/500 ms, matrix size 256×256, slice thickness = 3/0 mm, field of view = 256 mm) were acquired 8 minutes following administration of contrast agent (Magnevist, 0.2 ml/kg IV, Bayer, Wayne, NJ, USA).

**Figure 1:**
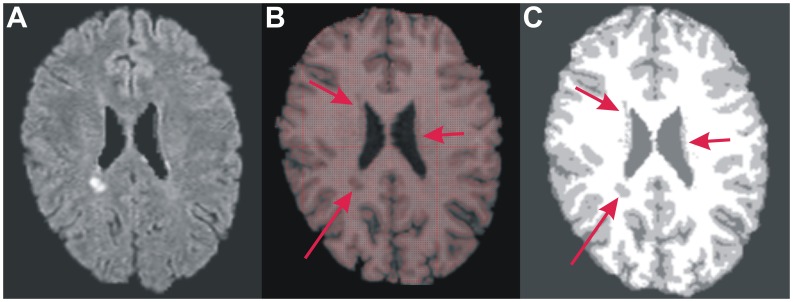
An example of automated volumetric assessment. A, FLAIR image from a patient with the usual extent of T2 hyperintense lesions seen in patients with clinically isolated syndrome. B, T1-weighted image with hypointense T1 lesions (arrows, lesion volume = 0.3 cm^3^). It is apparent that the T1 lesions were included in calculation of the overall brain volume (red area). C, T1-seighted image segmented by SIENAX. The T1 hypointense lesions (arrows) were misclassified into grey matter.

Automated image analysis was performed in the Department of Radiology, General University Hospital with our own software (ScanView.CZ by JK, www.scanview.cz). [10], [11] After standard image processing, signal intensity was normalised (peak = 10,000, white matter = 5000 artificial units). Overall volume of T2 lesions (T2LV) was measured in homogenised and filtered FLAIR images as the area exceeding the intensity of 140% of the white matter (WM) and the size of 11 voxels (i.e. a sphere with a 3 mm diameter). Lesion activity was evaluated in the T1-weighted images after contrast administration. The number of gadolinium-enhancing (Gd+) lesions was counted by a single experienced assessor (MV). Non-normalised brain volume was measured in T1-weighted images thresholded at above 4000 artificial units (Figure 1). Non-normalised grey matter (GM) and WM volumes were measured in T1-weighted images segmented with SIENAX automated image segmentation tool, version 2.60 (http://www.fmrib.ox.ac.uk/analysis/research/siena/). Despite the fact that no lesion inpainting was used, the T1 hypointense lesions were not excluded from the area outlined by ScanView.CZ as the brain tissue (see Figure 1). In contrast, the T1 lesions were misclassified by SIENAX as GM. The error introduced by this misclassification was 0.14±0.18% for the GM volumetry and 0.15±0.2% for the WM volumetry. The cross-sectional area of the corpus callosum (CC) was measured and averaged in seven 3-dimensional reconstructions of T1-weighted sagittal slices per patient (thickness = 1/0 mm) using an automated procedure. [10] For brain, GM and WM volumes and CC area, percentage changes relative to the baseline measurement in each patient were used in all analyses. In all instances, re-test and inter-rater errors were below 0.5% and 0.3%, respectively.

### Statistical Analysis

Statistical analyses were carried out with Statistica 10 (Statsoft, Tulsa, OK, USA) and R (http://www.R-project.org). To adjust for multiple hypothesis testing, all p-values were adjusted with Benjamini-Hochberg correction. Distribution of T2LV was normalised using logarithmic transformation.

Baseline demographic, clinical and MRI variables were compared between the CIS and the CDMS groups using Student’s t-test, Mann-Whitney U test or χ^2^ test, with mean differences in continuous variables quantified with Cohen’s d statistic. Logistic regression models adjusted for age, gender and changes in treatment were used to assess correlation between the volumetric MRI parameters at 2 years and CDMS conversion. Similar models were used to evaluate prediction of CDMS conversion by baseline T2LV and cumulative Gd+lesion number, brain, GM and WM volume changes as well as CC area change at 6 months. Optimal thresholds for the predictor variables were determined with receiver operating characteristic (ROC) curves. Repeated measures of clinical and MRI parameters were compared between the patient sub-groups (i.e. CIS vs. CDMS sub-groups or predictor sub-groups based on decrease in CC area or baseline T2LV) with mixed-effect models adjusted for patient age, gender and treatment changes. Cumulative risks of CDMS in the prediction sub-groups were evaluated with Cox proportional hazards models adjusted for patient age, gender and treatment changes. Since measures of CC area at 6 months were missing in 8 patients, analyses involving this parameter were carried out in 209 patients. Correlation between the MRI predictors was tested with linear regression. Associations between the number of relapses during the 2-year follow-up (categorised) and changes in clinical and MRI parameters were tested with linear or Poisson regression models (the latter was used to model cumulative Gd+lesion number) adjusted for sex, age, baseline EDSS and time from study enrolment. All models were fitted based on Akaike information criterion and Hosmer-Lemeshow χ^2^ test as appropriate.

## Results

Of the 217 patients included in this analysis, 92 (42%) converted to CDMS during the 2 years from the initial demyelinating event. An overview of the basic demographic, clinical and volumetric MRI parameters in the CIS and CDMS sub-populations is shown in Table 1. On average, the CDMS patients were younger with higher baseline T2 lesion volume and with more frequent Gd+lesions than the CIS patients (p≤0.02, t- or χ^2^ tests).

**Table 1:** Demographic, clinical and MRI characteristics of the sample.

		stable CIS	progressing to CDMS	Cohen d	p value*
**Subjects, number (females, %)**		125 (79, 63%)	92 (66, 72%)		NS
**Age, years ± SD**		30±8	28±8	0.35	0.01
**Time from the first event, months ± SD**		0.9±0.7	1.1±0.8	0.25	NS
**EDSS, median (interquartile range)**		1.5 (1.5, 2)	1.5 (1.5, 2)		
**MSFC, z ± SD**		0.36±0.53	0.22±0.54	0.26	NS
**T2 lesion volume, cm^3^± SD**		2.9±4.0	4.4±5.5	0.32	0.02
**Gd+lesions, patients**	**[0]**	108 (86%)	60 (65%)		
	[**1**]–[**2**]	13 (10%)	19 (21%)		
	**[3+]**	4 (3%)	13 (14%)		0.002
**Brain volume, cm^3^± SD**		1182±111	1169±115	0.12	NS
**GM volume, cm^3^± SD**		605±52	606±58	0.01	NS
**WM volume, cm^3^± SD**		547±57	539±56	0.15	NS
**Corpus callosum area, cm^2^± SD**		4.4±0.6	4.3±0.6	0.16	NS

* t-test, Mann-Whitney U or χ2 tests; T2 lesion volumes and cumulative number of Gd+lesions were compared after logarithmic transformation.

CDMS, clinically definite multiple sclerosis; CIS, clinically isolated syndrome; EDSS, Expanded Disability Status Scale; Gd+, gadolinium positive; GM, grey matter; MSFC, Multiple Sclerosis Functional Composite; NS, not significant; SD, standard deviation; WM, white matter.

### MRI and Clinical Parameters in CIS and CDMS

**Figure 2:**
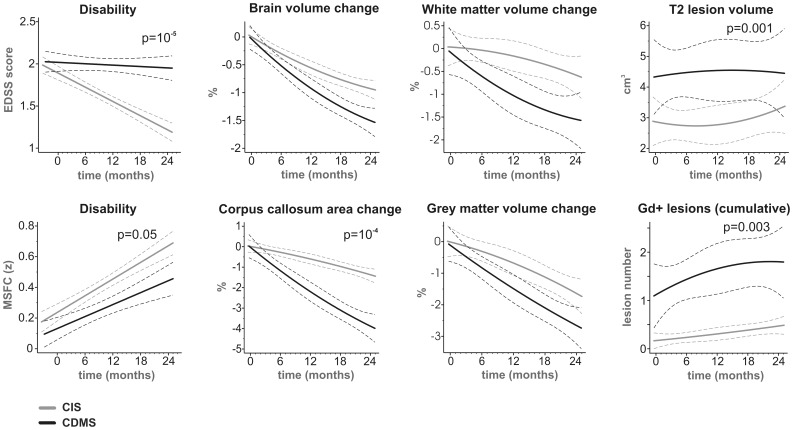
Disability and volumetric MRI parameters in patients with CIS and in those converting to CDMS. Dashed lines delineate 95% confidence intervals. Statistically significant p-values are shown. CIS, clinically isolated syndrome; CDMS, clinically definite multiple sclerosis; EDSS, Expanded Disability Status Scale; Gd+, gadolinium positive; MSFC, Multiple Sclerosis Functional Composite.

Figure 2 demonstrates clinical and MRI parameters recorded during the 2-year follow-up period. Both EDSS and MSFC indicated that neurological function recovered to a greater extent in the CIS than the CDMS group (p = 10^−5^ and 0.05, respectively, mixed model). While relative changes in whole brain, WM and GM volumes showed consistent trends to decrease faster in the CDMS group, these trends did not reach the level of statistical significance (p>0.1, mixed models). However, decrease in CC area was significantly more pronounced in the CDMS group than in the CIS group (approximately −3.9% vs. −1.3% over 2 years, respectively; p = 10^−4^, mixed model). The T2LV and cumulative Gd+lesion number were higher in the CDMS group compared to the CIS group throughout the study (p≤0.001, mixed models). The logistic model including all of the studied volumetric MRI parameters at 2 years identified two parameters independently correlated with CDMS conversion: the change in CC area (b = 0.2, SEM = 0.08, p = 0.008) and the overall number of MRI scans with Gd+lesions (b = 1.8, SEM = 0.7, p = 0.03). Interestingly, the model did not find any statistically significant association with cumulative number of Gd+lesions (p = 0.2).

### Predictors of CDMS Conversion

The logistic model identified CC area change at 6 months (b = 0.4, SEM = 0.1, p = 0.008) and baseline T2LV (b = 0.6, SEM = 0.2, p = 0.02) as being independent predictors of CDMS conversion within 2 years of the first demyelinating event. Linear regression analysis found only a weak correlation between these two predictors (r = 0.22, p = 0.004).

For CC area, a decrease of 1% over the first 6 months was identified as the best discriminator between the CIS and the CDMS groups at 2 years (area under ROC curve = 0.67). Of the patients with a 6-month decrease in CC area ≥1% vs.<1%, 69% vs. 41% reached CDMS, respectively (hazard ratio = 1.8, 95%CI = 1.1–2.9). A detailed account of the 2-year relative risks of CDMS conversion is given in Table 2. Moreover, those with a faster CC area decrease showed marginally more severe disability (identified by EDSS), accelerated decrease in whole brain and GM volume as well as higher T2LV and cumulative Gd+lesion number than those with less marked CC atrophy (p≤0.008, mixed models; see Figure S1).

**Table 2:** Relative risk of conversion to CDMS predicted by decrease in CC area at 6 months of CIS.

Decrease in corpus callosum area	Progression to CDMS		Odds ratio (95%CI)
	No	Yes	
0%	58	18	N/A*
0–0.5%	20	11	1.8 (0.7–4.4)
0.5–1%	13	12	3 (1–8.7)
1–2%	15	20	4.3 (1.5–12)
≥2%	16	25	5 (1.8–14)

* reference for the odds ratio estimates.

CC, corpus callosum; CDMS, clinically definite multiple sclerosis.

Similarly, baseline T2LV of 5 cm^3^ discriminated between the CIS and the CDMS groups at 2 years (area under ROC curve = 0.60). Here too, higher conversion rate was seen in patients with a baseline T2LV ≥5 cm^3^ (63%) than in those with a lower baseline T2LV (34%; hazard ratio = 2.5, 95%CI = 1.5–4). Also, marginally more severe disability (identified by MSFC), faster decrease in whole brain and WM volume, accelerated CC atrophy as well as higher cumulative Gd+lesion number were associated with high baseline T2LV (p≤0.05, mixed models; see Figure S2).

**Figure 3:**
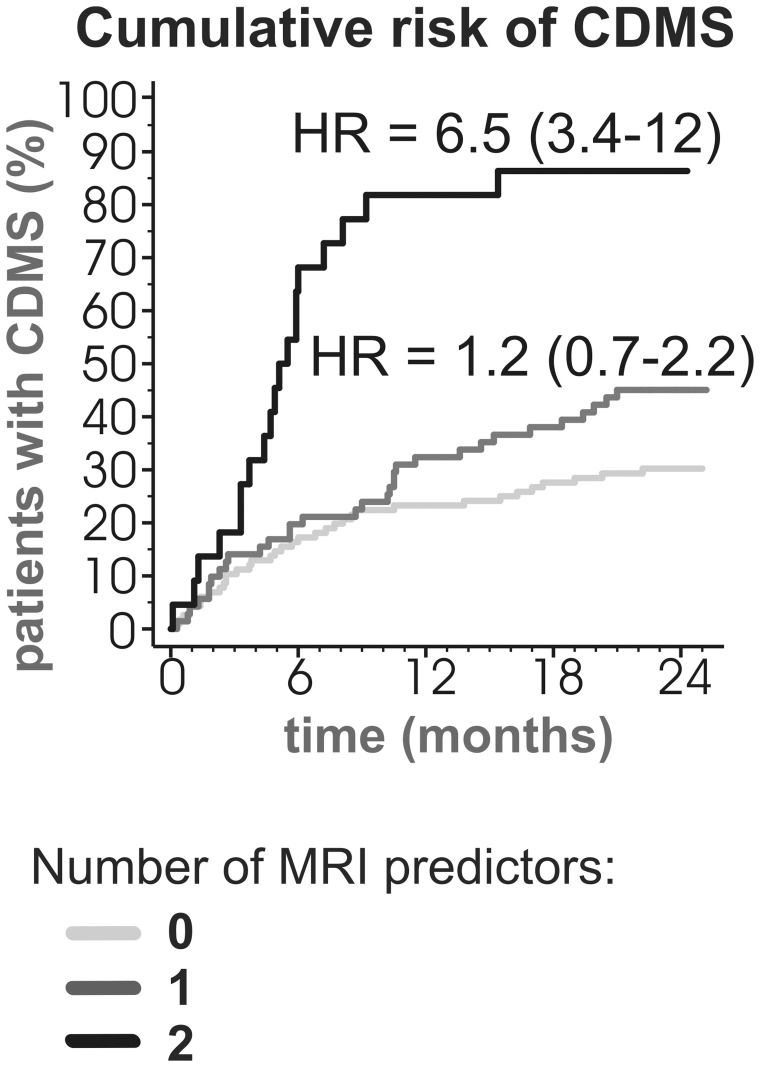
Cumulative risk of CDMS by number of volumetric MRI predictors. The MRI predictors found to be statistically significant by the logistic model were tested. These comprised decrease in corpus callosum area at 6 months ≥1%, and baseline T2 lesion volume ≥5 cm^3^. Hazard ratios with 95% confidence intervals are shown. CDMS, clinically definite multiple sclerosis, HR, hazard ratio.

Of the 116 patients fulfilling neither of the predictive criteria (i.e. decrease in CC area at 6 months ≥1% or baseline T2LV ≥5 cm^3^), 35 (30%) converted to CDMS during the 2-year follow-up period (Figure 3). In the sub-group fulfilling one of these criteria (71), 32 (45%) converted to CDMS (hazard ratio = 1.2, 95%CI = 0.7–2.2). Among the 22 patients fulfilling both predictive criteria, 19 (86%) reached CDMS (hazard ratio = 6.5, 95%CI = 3.4–12). The risk of CDMS in the sub-group fulfilling both predictive criteria was significantly higher than in the sub-group fulfilling only one criterion (hazard ratio = 4.7, 95%CI = 2.5–8.7).

### Effect of Relapses on MRI and Clinical Parameters

**Figure 4:**
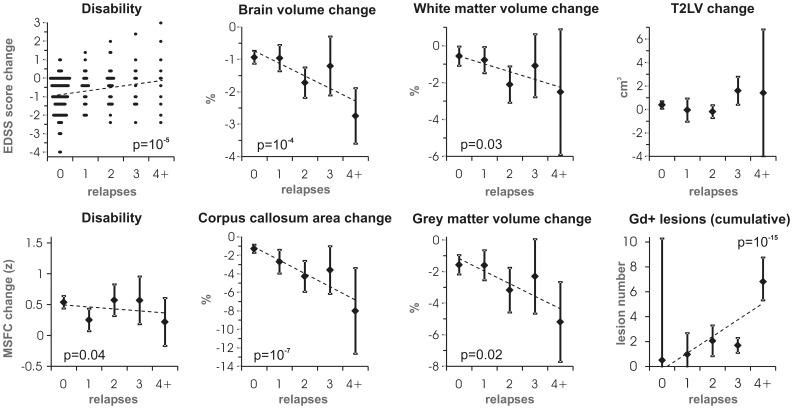
Effect of relapses on disability change and volumetric MRI parameters at 2 years of CIS. Patients were categorised by the number of relapses during the 2-year follow-up period (0: n = 125, 1: n = 30, 2: n = 35, 3: n = 16, 4+: n = 11). Least-squares regression lines (dashed) and statistically significant p-values are shown. EDSS, Expanded Disability Status Scale; Gd+, gadolinium positive; MSFC, Multiple Sclerosis Functional Composite; T2LV, T2 lesion volume.

We compared 2-year changes in clinical and MRI parameters between patients categorised by number of relapses (Figure 4). Smaller relapse numbers were associated with more pronounced reduction of disability (EDSS p = 10^−5^, MSFC p = 0.04, linear regression). Two-year changes in disability in the sub-groups with 0 vs. 4+relapses reached −1 (−1.5–0) vs. 0 (−1.5–1) for EDSS [median (interquartile range)] and 0.5 (0.4–0.6) vs. 0.2 (−0.2–0.6) for MSFC [mean (95%CI)], respectively. The rates of whole brain, GM and WM atrophy were proportional to the number of relapses (p≤0.03, linear regression). Moreover, similar but stronger association was apparent for the decrease in CC area, which reached 2-year averages of 1.3% (0.9–1.7%) vs. 8% (3.4–13%) in patients with 0 vs. 4+relapses [mean (95%CI)]. Finally, higher number of relapses was related to higher cumulative number of Gd+lesions (p = 10^−15^, Poisson regression). In contrast, no statistically significant relationship between the number of relapses and changes in T2LV was found (p = 0.6, linear regression).

## Discussion

We have shown here, in a cohort of 217 patients with CIS treated with interferon β, that the patients reaching CDMS within the initial 2 years of the first demyelinating event show poorer recovery of neurological function, more pronounced brain atrophy and higher overall inflammatory activity within the brain. Moreover, we have identified a 6-month decrease in the corpus callosum cross-sectional area and baseline T2LV as predictors of CDMS conversion. Finally, we have demonstrated that capacity to recover from disability diminishes and brain atrophy increases with the increasing number of early relapses.

It is evident that conversion to CDMS occurs predominantly during the first year of CIS, with a subsequent decline in its incidence (see Figure 3).[1]–[4], [12], [13] Overall, we have observed a 2-year CDMS conversion rate of 42%. This is in agreement with the reported conversion rates of 33–53% within 1–3 years following the first demyelinating event, [5], [14], [15] but relatively higher compared to the conversion rates reported in patients treated with interferon β (31% over 2 years). [5] This discrepancy could be attributed to our stringent inclusion criteria (abnormal MRI and CSF findings), which are known to improve diagnostic accuracy for MS in CIS and are suggestive of higher baseline disease activity. [16]

Similar to our study, other works have previously shown accelerated annual brain atrophy in patients converting to CDMS compared to those with no further relapses (0.5% vs. 0.26%, respectively). [4], [14] According to our current findings, atrophy of the CC shows even better correlation with the incidence of CDMS than global measures such as overall brain atrophy, GM and WM atrophy, and can therefore be seen as a more sensitive indicator of disease activity in CIS. In fact, CC pathology is associated with higher relapse rate (r = 0.35). [17] These observations are also concordant with the reported associations between disruption of the CC tracts and physical disability (r = 0.46) [17] and cognitive impairment (r = 0.31−0.66). [18] It can therefore be speculated that CC atrophy is determined by the extent of changes in other areas of the brain interconnected via interhemispheric projections.

It is known that abnormal brain MRI at the time of CIS is predictive of higher risk of CDMS conversion, ongoing disease activity and faster accumulation of disability. [2], [4], [8], [12], [19]–[22] Numbers of hyperintense T2 lesions and Gd+lesions at baseline were shown to predict the risk of CDMS, [1], [7] with 5-year CDMS conversion rates being 9%, 44% and 61% among patients fulfilling 0, 1–2 and 3–4 Barkhof criteria, respectively. [2] We were able to confirm the predictive value of T2 lesions, by identifying baseline T2LV as an independent predictor, but not of the incidence of Gd-enhancing lesions. It is worth noting that our analysis of the descriptive value of the volumetric MRI markers demonstrated that not the cumulative number of Gd+lesions but their presence/absence during the 2-year follow-up period was associated with CDMS conversion. This is in agreement with the fact that one additional clinical relapse is sufficient to define CDMS in patients with CIS and that this might be triggered by a single active lesion. Rather than to incidence of a clinical relapse, the number of Gd+lesions is likely to be related to relapse severity. [23]

In addition to baseline T2LV, Di Fillipo and colleagues have shown that brain atrophy at 1 year of CIS also predicts CDMS conversion. [4] Here too, our results suggest that CC atrophy is a more sensitive marker of disease activity than the global atrophy measures: unlike the global measures, it predicts CDMS conversion as early as 6 months of CIS. We have tested cut-off values for dichotomised predictors of CDMS risk: 5 cm^3^ for the baseline T2LV and 1% for CC atrophy at 6 months, with the respective hazard ratios being 2.5 and 1.8. The latter is in agreement with our previous report of 2% cut-off value for CC atrophy at 1 year of treatment initiation to predict accumulation of physical disability in relapsing-remitting MS. [10] Most importantly, the two early MRI predictors identified in our study are complementary, as shown by the CDMS conversion rate, which was significantly higher in patients fulfilling both criteria (86%) than in those fulfilling only one criterion (45%, hazard ratio = 4.7). This observation is in agreement with the hypothesis that brain atrophy and the extent of inflammatory lesions should be seen as markers of distinct pathophysiological mechanisms. [24]

Interestingly, in our analysis, GM atrophy was not found to be significantly different between the CIS and the CDMS patients, neither was it identified as a predictor of CDMS conversion. This is in agreement with the outcomes of recent works, which demonstrated selective regional but not global GM atrophy in CIS [25], [26]. Thus the global GM atrophy is thought to be minimal in this early disease stage, and therefore it is unlikely that its increment should be differential in the sub-population with sustained CIS and that with imminent conversion to CDMS.

There has been ongoing discussion concerning the effect of relapses on the progression of MS. [27] Here, we have shown that higher relapsing activity is associated with more prominent inflammatory activity within the brain (marked by the incidence of Gd+lesions), poorer recovery from disability, and with faster progression of brain atrophy (best marked by CC atrophy) within 2 years of CIS. It is therefore possible that early in the disease, relapses drive a decrease in the functional reserve of the CNS and thus contribute to irreversible CNS damage. [28] Alternatively, relapses may be collinear with disability progression and the accumulation of MRI pathology, all of which may represent parallel outcomes of common underlying pathophysiological mechanisms. In any case, our observations are in agreement with other works, which have shown an effect of early but not late relapsing activity on disability.[29]–[31] Therefore, timely treatment of relapses is likely to be crucial for the amelioration of future disability. [32]

Despite the fact that patients with higher baseline T2LV were more prone to ongoing relapsing activity (CDMS), changes in T2LV were not significantly associated with relapse frequency. This implies that T2LV may merely be a marker of duration and activity of MS in its sub-clinical stage. In contrast, incidence of Gd+lesions was related to the number of reported relapses. Therefore, it could be speculated that the frequency of inflammatory events within the CNS does not necessarily lead to an increase in the overall T2LV. Lessunder, the number of lesions and overall lesion volume may represent two distinct parameters with different descriptive and predictive values. [11]

Our current analysis was carried out in a homogeneous sample of early-presenting CIS patients, in whom the risk of imminent MS was greatly enhanced by positive MRI and CSF findings. Due to these criteria and also the fact that majority of our patients were followed in MS centres our sample may not be representative of the general CIS population. Even though 2-year follow-up was sufficient for evaluation of CDMS conversion (as most of CDMS conversions occur within the initial year of CIS), it was insufficient to detect significant accumulation of disability in CIS quantified by either EDSS or MSFC. Therefore, we have omitted the predictive analysis of disability severity. Another limitation of our study was the absence of T2 lesion counts, which were not carried out as volumetric measures were used instead. Finally, an error was introduced to our quantification of GM and WM volumes by misclassification of hypointense T1 lesions into the GM. Further studies adjusting for this misclassification should determine the evolution of GM and WM atrophy in CIS more accurately.

It had been previously reported that early disease modifying treatment slows progressive loss of brain tissue and reduces conversion to CDMS in patients with CIS. [5] In the present study, we have identified two volumetric MRI markers, which are suitable complementary estimators of individual relative risk of CDMS conversion.

### Notes

* All CSF samples were analysed by one accredited laboratory (Department of Biochemistry, General University Hospital in Praha).

## Supporting Information

Figure S1 **Clinical and MRI parameters in patients with low and high corpus callosum area decrease.** Disability, time to CDMS and volumetric MRI parameters in patients with atrophy of the corpus callosum within 6 months of CIS<1% and ≥1%. 95% confidence intervals (dashed lines) and statistically significant p-values are shown. Data presented are from 209 patients with corpus callosum volumetry available at month 6. CDMS, clinically definite multiple sclerosis; Gd+, gadolinium positive; EDSS, Expanded Disability Status Scale; MSFC, Multiple Sclerosis Functional Composite
(TIF)

Figure S2 **Clinical and MRI parameters in patients with low and high baseline T2 lesion volume.** Disability, time to CDMS and volumetric MRI parameters in patients with baseline T2 lesion volume<5 cm3 and ≥5 cm3. 95% confidence intervals (dashed lines) and statistically significant p-values are shown. CDMS, clinically definite multiple sclerosis; EDSS, Expanded Disability Status Scale; Gd+, gadolinium positive; MSFC, Multiple Sclerosis Functional Composite; T2LV, T2 lesion volume
(TIF)
