# Supplementary figures and images for: Volumetric MRI Markers and Predictors of Disease Activity in Early Multiple Sclerosis: A Longitudinal Cohort Study
Source: PLoS One. 2012 Nov 15;7(11):e50101. doi: 10.1371/journal.pone.0050101 (PMC3499512; doi:10.1371/journal.pone.0050101)

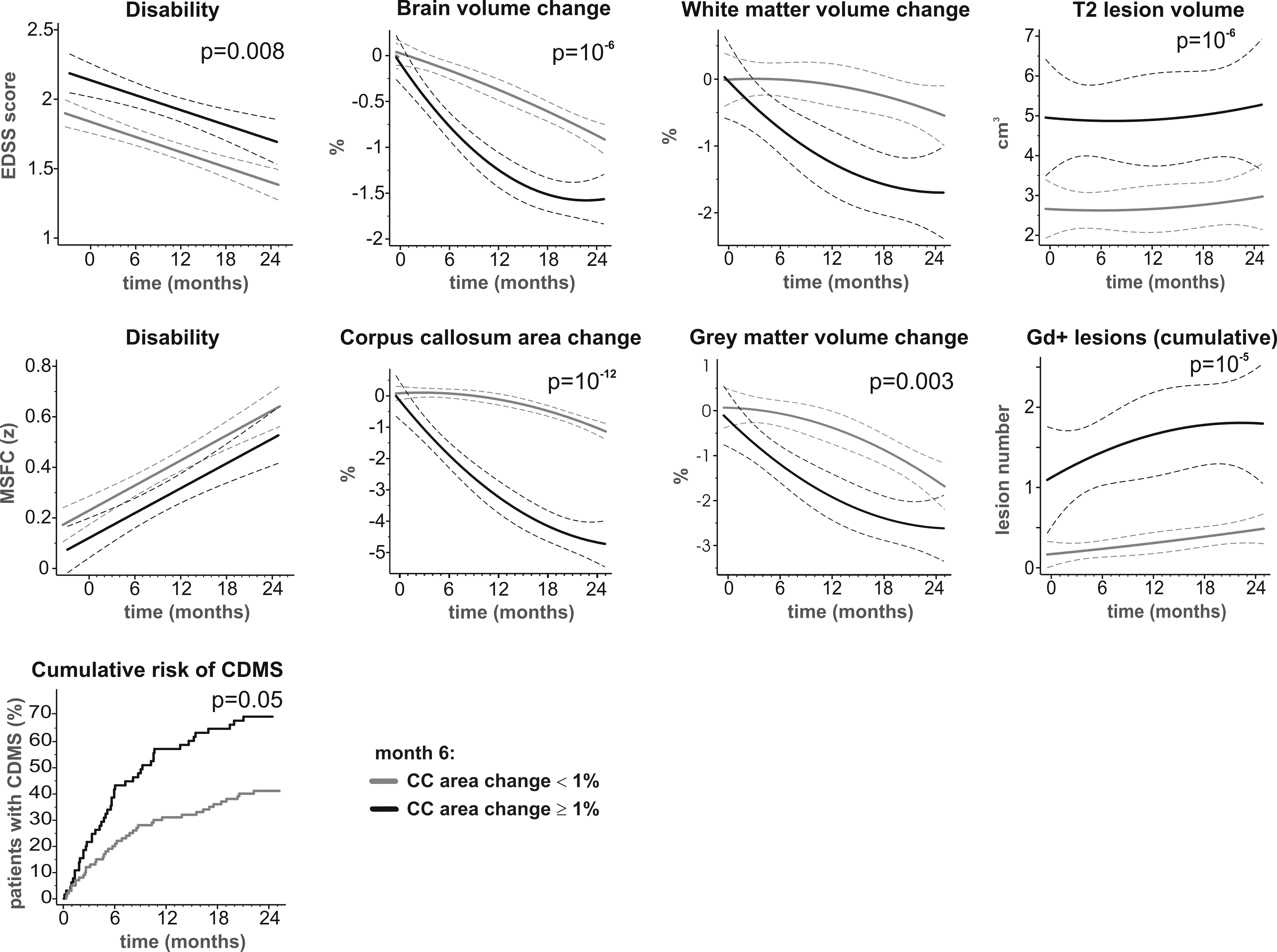

Supplement: Figure S1 — Clinical and MRI parameters in patients with low and high corpus callosum area decrease. Disability, time to CDMS and volumetric MRI parameters in patients with atrophy of the corpus callosum within 6 months of CIS<1% and ≥1%. 95% confidence intervals (dashed lines) and statistically significant p-values are shown. Data presented are from 209 patients with corpus callosum volumetry available at month 6. CDMS, clinically definite multiple sclerosis; Gd+, gadolinium positive; EDSS, Expanded Disability Status Scale; MSFC, Multiple Sclerosis Functional Composite (TIF) [file pone.0050101.s001.tif]

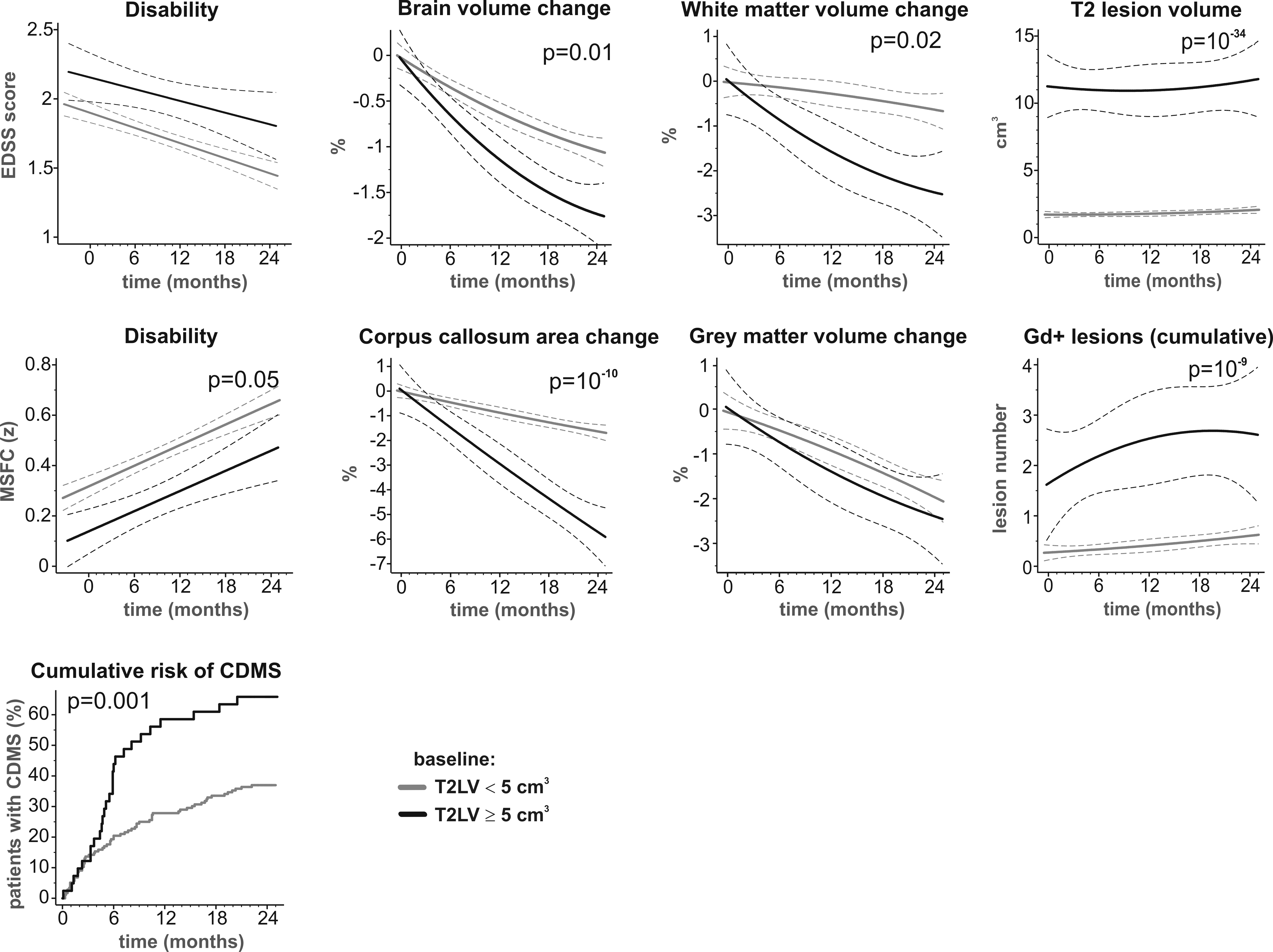

Supplement: Figure S2 — Clinical and MRI parameters in patients with low and high baseline T2 lesion volume. Disability, time to CDMS and volumetric MRI parameters in patients with baseline T2 lesion volume<5 cm3 and ≥5 cm3. 95% confidence intervals (dashed lines) and statistically significant p-values are shown. CDMS, clinically definite multiple sclerosis; EDSS, Expanded Disability Status Scale; Gd+, gadolinium positive; MSFC, Multiple Sclerosis Functional Composite; T2LV, T2 lesion volume (TIF) [file pone.0050101.s002.tif]
